# Measure of energy related biochemical metabolites changes during peri-partum period in Makouei breed sheep

**Published:** 2016-03-15

**Authors:** Vahid Mohammadi, Ehsan Anassori, Shoja Jafari

**Affiliations:** 1 *Department of Internal Medicine and Clinical Pathology, Faculty of Veterinary Medicine, Urmia University, Urmia, Iran; *; 2 *West Azerbaijan Jihad-Agriculture Organization, Makouei Sheep Breeding Station, Maku, Iran.*

**Keywords:** Beta-hydroxybutyrate, Blood metabolites, Makouei sheep, Non- esterified fatty acids

## Abstract

Makouei sheep is one of the famous breeds in Iran which is reared in Azerbaijan province for their meat, milk and wool. Fifty clinically healthy Makouei ewes were selected to study the variations in energy-related blood metabolites during peri-partum period. Blood was collected from Jugular vein from each sheep on day 7 before the expected lambing time, day of parturition and also day 7 postpartum to determine total protein, albumin, urea, cholesterol, glucose, triglyceride, β-hydroxybutyrate (BHB) and non- esterified fatty acid (NEFA) levels. Serum total protein and albumin concentrations were gradually decreased during pre-partum period and reached the lowest level after parturition (*p* > 0.05). Blood urea concentration was significantly decreased to the lowest level at parturition (*p* < 0.05). Serum cholesterol and triglycerides were gradually decreased and reached low levels after lambing (*p* < 0.05). Serum glucose concentrations were significantly lower at pre-partum period than post-partum (*p* < 0.05). The serum NEFA and BHB concentrations were higher before lambing and thereafter decreased (*p* < 0.05). Current findings regarding the blood parameters may expand our knowledge for the diagnosis and prognosis of reproductive and metabolic diseases in Makouei sheep during these phases.

## Introduction

Makouei sheep is one of the famous breeds in Iran which is reared in Azerbaijan province with an approximate population size of 2.70 million.^1^ Makouei is a multipurpose sheep which its main products are meat, milk and wool. The Makouei sheep is one of the most popular breeds for milk production and is well adapted to different conditions and can be successfully reared in different regions of the world. The lactation period is 120 days and average milk yield is 75.00 kg.^2^ Pregnancy and lactation are physiological states considered to modify metabolism in animals and induce stress.^3^^,^^4^ Hematological and biochemical changes have been studied in different breeds of sheep and goats.^5^^,^^6^ During lactation and advanced pregnancy in ruminants, maternal stores are involved in providing energy for production processes, which may affect blood serum chemistry values. Combination of these events with a decrease in feed intake, create negative energy balance.^7^ Generally, the peri-partum period is characterized by negative energy balance, body fat mobilization, and the elevation of circulating non-esterified fatty acids (NEFA) and ketone bodies.^8^ Therefore, the identification of metabolic changes in Makouei sheep in this period and prediction of some metabolic disorders, such as pregnancy toxemia, fatty liver and production ketosis, would be economically beneficial. 

During lactation, the mammary gland secretory cells utilize 80.00% of the blood-circulating metabolites for milk synthesis. Synthesis and composition of the milk depend on the speedy infiltration of precursors of milk compounds, including free amino acids, glucose and fatty acids into the mammary gland cells. Among those precursors of milk, protein, lactose and fat are produced by mammary gland cells. Changes in milk composition and decrease in milk production have been reported for decrease in the production of protein, lactose or fat.^9^

During lactation and advanced pregnancy in ruminants, the insulin concentration in blood and responsiveness of glucose reserves to insulin decrease. In this case, fat mobilization enhances to obtain free fatty acids from adipose tissue as an alternative energy resource. An increase in free fatty acids concentrations occurs due to a decreased insulin concentration.^7^ The identification of changes in the meta-bolism of such sheep in various reproduction phases, the determination of abnormal metabolic states and the prediction of some metabolic disorders such as pregnancy toxemia and fatty liver could ultimately provide some advantages to producers. 

To the best of our knowledge, there are no published data on the variations of energy-related metabolites around parturition and the impact of lactation on the parameters in Makouei sheep. 

The objective of this study was to evaluate the rate of negative energy balance (NEB) in peri-parturient Makouei sheep to understand the variations of energy-related metabolites during this critical period.

## Materials and Methods

This study was carried out at the Makouei Sheep Breeding and Raising Station (MSBS) in Maku, Western Azerbaijan, Iran. From the flock at the station, 50 estrus synchronized clinically healthy 2- to 5-year-old Makouei ewes, with a body weight of 45.00 ± 2.30 kg (ranging from 39.00 to 47.00 kg) and parity ranging between two to four lambing were used in the trial. Ewes were assigned as twin-bearing ewes following ultrasonography 35 days after synchronized mating. The mean birth weight of the lambs was 3.80 ± 0.50 kg (ranging from 3.20 to 4.10 kg). The animals were housed in a well-ventilated building with free access to water. The trial lasted for the period between seven days of pre-partum and seven days of post-partum. Two weeks were served as an adaptation period before the main experiments started, allowed the ewes to adapt to the new ration and housing conditions. Diets were formulated based on NRC guidelines, offered as a total mixed ration (TMR), and adjusted for changes in dry matter intake (DMI) weekly.^8^ Total mixed ration offered *ad libitum* twice daily at 09:00 AM and 15:00 PM. Nutrient composition of the TMR was varied with the stage of pregnancy and lactation (Table 1). 

The concentrate to roughage ratio of the TMR was 40:60 for pre- and post-partum period. The blood samples were obtained from each animal one week before parturition, day of parturition and one week after parturition via jugular vein.

**Table 1:** Ingredients and chemical composition of diets in pre-parturition period (two weeks before parturition) and post-parturition period (two weeks after parturition

**Parameters**	**Pre-partum**	**Post- partum**
**Dry matter intake (kg day** ^-1^ **)**	1.16	1.58
***Ingredients (% DM):***		
**Alfalfa hay **	26.00	26.00
**Corn silage**	30.00	30.00
**Wheat straw **	4.50	4.50
**Barley grain **	25.00	28.50
**Soybean meal **	4.50	4.50
**Wheat bran **	8.50	4.70
**Di-Calcium phosphate**	0.50	0.32
**Sodium bicarbonate **	-	0.50
**Salt**	0.50	0.50
**Vitamin and mineral premix**	0.50	0.50
***Chemical composition:***		
**Metabolizable energy (Mcal day** ^-1^ **)**	2.81	3.40
**Net energy (Mcal kg** ^-1^ **)**	1.55	1.55
**Crude protein (% DM) **	14.5	14.2
**Acid detergent fiber (% DM) **	28.4	27.9
**Neutral detergent fiber (% DM)**	40.3	39.7
**Ether extract (% DM) **	2.40	2.30
**Calcium (% DM) **	0.62	0.57
**Phosphorus (% DM) **	0.39	0.35

DM: Dry matter

The samples were kept at room temperature for 20 min, and then centrifuged at 3000 rpm for 10 min. The serum samples were stored at − 20 ˚C until analysis. The concentrations of β-hydroxybutyrate (BHB) and non-esterified fatty acids (NEFA) were determined using D-3-hydroxybutyrate kit and a NEFA Kit (Randox Laboratories Ltd., Ardmore, UK), respectively. Serum urea, total protein, albumin, triglyceride and blood glucose concentrations were determined colorimetrically using commercial kits (Pars Azmoon Co., Tehran, Iran). An automated biochemical analyzer (Model RA-1000; Technicon Instruments Corp., Tarrytown, USA) was used for all measurements.^11^

Measurement of data accuracy was performed using control serum (Randox control sera, Antrim, UK). Mean values and standard errors were calculated for each parameter. Data were analyzed using a Repeated Measures ANOVA in SPSS (Version 11; SPSS Inc., Chicago, USA). The differences were set at *p *< 0.05.

## Results

In the current study all the blood parameters were within or close to normal range values for healthy ewes.^9^ The serum metabolite concentrations during the peri-partum period are shown in Table 2. The serum NEFA and BHB concentrations were higher before lambing; thereafter decreased (*p* < 0.05). Serum glucose concentrations were significantly lower in pre-partum period than post-partum (*p* < 0.05). Serum cholesterol and triglycerides were gradually decreased after lambing (*p* < 0.05). Serum total protein and albumin concentrations were numerically decreased during pre-partum period and reached the lowest level after parturition (*p* > 0.05). Blood urea concentrations were significantly decreased at parturition (*p* < 0.05). 

**Table 2.** Biochemical parameters in Makouei sheep at pre-partum (one week before), parturition and post-partum (one week after) periods. Data are presented as mean ± SE

**Parameter**	**Pre-partum**	**Parturition**	**Post-** **partum**
**Glucose (mg dL** ^-1^ **)**	78.72 ± 2.08^b^	82.02 ± 1.47^b^	88.74 ± 1.00^a^
**Triglyceride (mg dL** ^-1^ **)**	46.54 ± 1.45^a^	35.38 ± 1.12^b^	21.12 ± 0.58^c^
**Cholesterol (mg dL** ^-1^ **)**	56.70 ± 1.19^a^	47.78 ± 0.92^b^	47.30 ± 0.79^c^
**Urea (mg dL** ^-1^ **)**	44.60 ± 0.96^a^	20.56 ± 0.57^b^	21.22 ± 0.54^c^
**Total protein (g dL** ^-1^ **)**	7.00 ± 0.35	6.96 ± 0.32	6.32 ± 0.02
**Albumin (g dL** ^-1^ **)**	4.91 ± 0.05	4.32 ± 0.04	3.96 ± 0.03
**Non-esterified fatty acids (mmol L** ^-1^ **)**	0.42 ± 0.03 c	0.49 ± 0.04^a^	0.46 ± 0.03^b^
**β-hydroxybutyrate (mmol L** ^-1^ **)**	0.87 ± 0.04^a^	0.75 ± 0.03^b^	0.60 ± 0.03^c^

abc Different superscripts indicates significant differences in the same row (*p* < 0.05).

## Discussion

Glucose concentrations in the present study were in accordance with previous experimental studies that recorded serum glucose levels to be higher in lactation than pregnancy in ewes.^12^^-^^16^ The increase may reflect the recovery of feed intake and improving energy status of the ewe after lambing. Negative energy balance appears to be related to the glucose demands of the fetal-placental unit in pregnant ewes. Approximately 60.00% of fetal growth is known to take place during the last six weeks of pregnancy and within this period the most important etiological factor for pregnancy toxemia is decline in the plane of nutrition.^9^ In this period, fetal growth is rapid and the demands for energy are markedly increased.^17^ Although glucose is the primary metabolic fuel and is absolutely essential for vital organ function, fetal growth and milk production,^18^ it is an insensitive measure of energy status, as it is subject to tight homeostatic regulation.^19^ Measuring serum BHB concentration may thus be served as a useful method in monitoring the energy status of pregnant ewes. Values of 0.80 to 1.60 mmol L^-1^ indicate a negative energy balance.^20^ In the present study, BHB concentrations were ranged from 0.60 to 0.87 mmol L^-1^ during pre- and post-parturition (normal level of BHB; 0.47 to 0.63 mmol L^-1^).^9^

NEFA reflects the magnitude of fat mobilization from fat stores in response to negative energy balance. The gradual increase of plasma NEFA during the final days of pre-partum period may be explained by the gradual depression of DMI observed during this time. This increase is due to the required energy for parturition and milk production.^18^ In the present study, elevated NEFA levels (ranged from 0.42 to 0.49 mmol L^-1^) during peri-partum period were consistent with normal levels (NEFA, < 0.45 mmol L^-1^) described by Kaneko.^19^ Several researchers reported higher blood cholesterol and triglyceride concentrations during late pregnancy in sheep.^3^^,^^14^^,^^24^^,^^25^ This increase during the last weeks of pregnancy appears to be related to the energy demands of the fetal-placental unit in pregnant ewes.^26^ Another explanation for an increase in the serum cholesterol concentration recorded in the late pregnancy compared to day seven post-partum may be due to insulin. Its response is significantly reduced in ewes during late pregnancy. The diminished response of the target tissue to insulin during late pregnancy predisposes the ewes to an increase in blood cholesterol and lipoprotein concentrations.^27^

Blood urea concentrations were at the lowest level at parturition. The decrease in serum blood urea nitrogen around parturition may be associated with the decline of feed intake due to stress and hormonal changes during lambing.^16^

Serum total protein concentration was numerically decreased at the time of parturition and one week after parturition. However, this was not significant. These results were in agreement with Taghipour *et al*.^16^ This decrease in serum total protein may be related to the fact that the fetus synthesizes all its proteins from the amino acids derived from the dam, and growth of the fetus is increased exponentially reaching a maximum level, especially in muscles, during late pregnancy.^28^ However, serum albumin concentrations were not significantly changed in the present study. It could be concluded that the globulins were responsible for variations of total proteins levels. Bayoumi *et al*. found decrease of serum total protein, mainly due to decrease in globulin, especially alpha 1 and gamma fractions. This was thought to be due to the production of globulin-rich colostrum. The ability to synthesize the constituents of milk appears in sheep three to four weeks before parturition. Drainage of globulins to mammary glands for colostrum synthesis may be considered as a main factor for serum total protein reduction.^29^ Concentrations of blood plasma metabolites have been commonly used to assess the plane of nutrition in ruminants and the results of this study showed that the animals had better energy status as indicated by lower BHB and NEFA compared to higher levels of these metabolites in metabolic disorders such as pregnancy toxemia. The lower NEFA values indicate that there is less fat mobilization in these animals,^30^ whereas, the higher cholesterol values suggest that there is greater lipoprotein export from the liver.^31^ On the other hand, the predicted requirements for energy and protein for Makouei ewes in the physiologically critical period of pre- and post-parturition seem to alleviate the negative energy balance. Therefore, adjusting the diet toward the end stages of pregnancy as well as simultaneously monitoring of BHB, NEFA and cholesterol would reduce the occurrence of pregnancy toxemia in Makouei ewes.

## References

[1] Abbasi MA, Ghafouri-Kesbi F (2011). Genetic co(variance) com-ponents for body weight and body measurements in Makooei sheep. Asian Aust J Anim Sci.

[2] Akbarinejad V, Kazempoor R, Shojaei M (2014). Atlas of Iranian Sheep Breeds.

[3] Nazifi S, Saeb M, Ghavami S (2002). Serum lipid profile in iranian fat‐tailed sheep in late pregnancy, at parturition and during the post‐parturition period. J Vet Med A Physiol Pathol Clin Med.

[4] Raoofi A, Jafarian M, Safi S (2013). Fluctuations in energy-related metabolites during the peri-parturition period in Lori-Bakhtiari ewes. Small Rum Res.

[5] Eshratkhah B, Forouzan V, Ahanpanjeh J (2011). Relationship between the level of plasma insulin and lipid profile in Iranian fat-tailed sheep. Com Clin Pathol.

[6] Vihan VS, Rai P (1987). Certain hematological and biochemical attributes during pregnancy, parturition and post parturient periods in sheep and goats. Ind J Anim Sci.

[7] Maas J, Pearson EG, Smith BP (2009). Hepatic lipidosis. Large animal internal medicine.

[8] NRC (2007). Nutrient requirements of small ruminants: Sheep, goats, cervids and new world camelids.

[9] Radostits OM, Gay CC, Hinchcliff KW (2007). Veterinary medicine.

[10] Hatfield PG, Head W, Fitzgerald JA (1999). Effects of level of energy intake and energy demand on growth hormone, insulin, and metabolites in Targhee and Suffolk ewes. J Anim Sci.

[11] Sadjadian R, Seifi HA, Mohri M (2013). Variations of energy biochemical metabolites in periparturient dairy Saanen goats. Comp Clin Path.

[12] Henze P, Bickhardt K, Fuhrmann H (1994). The influence of the hormones insulin, cortisol, growth hormone and total estrogen on the pathogenesis of Ketosis in sheep. Dtsch Tierarztl Wochenschr.

[13] Takarkhede R, Gondane V, Kolte A (1999). Biochemical profile during different phases of reproduction in ewes in comparison to rams. Indian Vet J.

[14] Balikci E, Yildiz A, Gürdogan F (2007). Blood metabolite concentrations during pregnancy and postpartum in Akkaraman ewes. Small Rum Res.

[15] Moghaddam G, Hassanpour A (2008). Comparison of blood serum glucose, beta hydroxybutyric acid, blood urea nitrogen and calcium concentrations in pregnant and lambed ewes. J Anim Vet Adv.

[16] Taghipour B, Seifi HA, Mohri M (2010). Variations of energy related biochemical metabolites during peri-parturition period in fat-tailed baloochi breed sheep. Iranian J Vet Sci and Tech.

[17] Moallem U, Rozov A, Gootwine E (2012). Plasma concentrations of key metabolites and insulin in late-pregnant ewes carrying 1 to 5 fetuses. J Anim Sci.

[18] Le Blanc S (2006). Monitoring programs for transition dairy cows.

[19] Kaneko JJ (1989). Clinical biochemistry of domestic animals.

[20] Herdt TH (2000). Variability characteristics and test selection in herd-level nutritional and metabolic profile testing. Vet Clin North Am Food Anim Pract.

[21] Edmondson MA, Pugh DG, Anderson DE, Rings DM (2009). Pregnancy toxemia in sheep and goats.

[22] Schlumbohm C, Harmeyer J (2004). Hyperketonemia impairs glucose metabolism in pregnant and nonpregnant ewes. J Dairy Sci.

[23] Bertics SJ, Grummer RR, Cadorniga-Valino C (1992). Effect of prepartum dry matter intake on liver triglyceride concentration and early lactation. J Dairy Sci.

[24] Al-Dewachi O (1999). Some biochemical constituents in the blood serum of pregnant Awassi ewes. Iraqi J Vet Sci.

[25] Hamadeh M, Bostedt H, Failing K (1996). Studies on relevant metabolism parameters in blood plasma of highly pregnant and nonpregnant ewes [German]. Berl Munch Tierarztl Wochenschr.

[26] Watson TDG, Burns L, Packard CJ (1993). Effects of pregnancy and lactation on plasma lipid and lipoprotein concentrations, lipoprotein composition and post-heparin lipase activities in Shetland pony mares. J Reprod Fertil.

[27] Schlumbohm C, Sporleder H, Gurtler H (1997). The influence of insulin on metabolism of glucose, free fatty acids and glycerol in normo-and hypocalcemic ewes during different reproductive states [German]. Dtsch Tierarztl Wochenschr.

[28] Jainudee MR, Hafez ESE, Hafez ESE (1994). Gestation, prenatal physiology and parturition. Reproduction in farm animals.

[29] Bayoumi MT, Assad F, Nassar AM (1986). Serum protein electrophoresis in different physiological stages in ewes. World Rev Anim Prod.

[30] Grummer RR (1993). Etiology of lipid-related metabolic disorders in periparturient dairy cows. J Dairy Sci.

[31] Kaneene JB, Miller R, Herdt TH (1997). The association of serum nonesterified fatty acids and cholesterol, management and feeding practices with peri-partum disease in dairy cows. Prev Vet Med.

